# Multicompartmental non-invasive sensing of postprandial lipemia in humans with multispectral optoacoustic tomography

**DOI:** 10.1016/j.molmet.2021.101184

**Published:** 2021-02-05

**Authors:** Nikolina-Alexia Fasoula, Angelos Karlas, Michael Kallmayer, Anamaria Beatrice Milik, Jaroslav Pelisek, Hans-Henning Eckstein, Martin Klingenspor, Vasilis Ntziachristos

**Affiliations:** 1 Technical University of Munich, School of Medicine, Chair of Biological Imaging, Germany; 2 Helmholtz Zentrum München, Neuherberg, Institute of Biological and Medical Imaging, Germany; 3 Clinic of Vascular and Endovascular Surgery, Klinikum rechts der Isar, Munich, Germany; 4 Department of Vascular Surgery, University Hospital Zurich, Zurich, Switzerland; 5 Chair of Molecular Nutritional Medicine, TUM School of Life Sciences Weihenstephan, Technical University of Munich, Freising, Germany; 6 EKFZ-Else Kröner-Fresenius Zentrum for Nutritional Medicine, Technical University of Munich, Freising, Germany; 7 ZIEL-Institute for Food &Health, Technical University of Munich, Freising, Germany

**Keywords:** Cardiovascular risk, Fat, Hyperlipidemia, Lipid metabolism, Metabolic imaging, Photoacoustics

## Abstract

**Objective:**

Postprandial lipid profiling (PLP), a risk indicator of cardiometabolic disease, is based on frequent blood sampling over several hours after a meal, an approach that is invasive and inconvenient. Non-invasive PLP may offer an alternative for disseminated human monitoring. Herein, we investigate the use of clinical multispectral optoacoustic tomography (MSOT) for non-invasive, label-free PLP via direct lipid-sensing in human vasculature and soft tissues.

**Methods:**

Four (n = 4) subjects (3 females and 1 male, age: 28 ± 7 years) were enrolled in the current pilot study. We longitudinally measured the lipid signals in arteries, veins, skeletal muscles, and adipose tissues of all participants at 30-min intervals for 6 h after the oral consumption of a high-fat meal.

**Results:**

Optoacoustic lipid-signal analysis showed on average a 63.4% intra-arterial increase at ~ 4 h postprandially, an 83.9% intra-venous increase at ~ 3 h, a 120.8% intra-muscular increase at ~ 3 h, and a 32.8% subcutaneous fat increase at ~ 4 h.

**Conclusion:**

MSOT provides the potential to study lipid metabolism that could lead to novel diagnostics and prevention strategies by label-free, non-invasive detection of tissue biomarkers implicated in cardiometabolic diseases.

## Highlights

• MSOT provides non-invasive and label-free imaging of postprandial lipid dynamics.
• MSOT provides high-resolution multicompartmental lipid visualizations *in vivo.*
• Clinical hand-held MSOT is a flexible and highly-portable molecular imaging tool.

## 1 Introduction

High blood lipid levels in either fasting or postprandial states indicate high risk for developing cardiovascular (CVD) and metabolic diseases, such as coronary artery disease (CAD), stroke, peripheral arterial disease (PAD), obesity, diabetes, and non-alcoholic fatty liver disease (NAFLD) [[1], [2], [3], [4], [5], [6], [7]]. Although fasting blood lipid measurements have been successfully employed over the past decades for disease risk stratification [8,9], the postprandial blood lipid levels are often better predictors of acute complications, such as heart attack, stroke, or death [10]. For example, an analysis of the lipid profiles in 42,710 patients in the non-fasting state showed that the non-fasting levels of lipids predicted increased risk of cardiovascular events [9]. Furthermore, lipid analysis of 8,270 subjects provided strong evidence to support the routine use of postprandial lipid levels in clinical practice for accurate risk assessment of atherosclerotic CVD [11].

Assessment of fasting blood lipid concentrations is primarily done by single time-point blood sampling via venipuncture. Moreover, measurements of blood lipid dynamics can be carried out by several blood samplings after the consumption of a high-fat meal to yield more information on lipid metabolism (postprandial lipid profiling, PLP) [12]. In both cases, the acquisition of blood samples causes patient discomfort, consumes hospital time and resources, and is only appropriate for infrequent sampling. Importantly, these methods of sampling lipids only allow observations in the blood and not in different tissue types.

Current non-invasive methods for lipid measurements in humans include breath measurements and eye image analysis. Lipid metabolism produces volatile organic compounds (VOCs, such as 2-pentyl nitrate, carbon dioxide, methyl nitrate, and toluene), some of which are exhaled. In one study, these VOCs were exploited to quantify blood lipid levels [13]. Lipids can also accrue in the cornea after arriving through the blood stream of the limbal vessels under hyperlipidemic conditions. Processing of images taken from the human eye (RGB color representation) calculated the corneal lipid deposition by analyzing the grayscale intensity level within the region of lipid deposits [14]. Based on these values and known lipid values in the blood, a regression model was developed and used to indirectly estimate the blood lipid levels from the extracted image parameters. Both non-invasive methods described above provide estimates of lipid concentration in blood based on mathematical models. However, neither of these methods offers a localized measurement of lipids in blood or tissues, nor direct correlation analysis with either measured VOCs or image intensities. For this reason, they have yet to be integrated into research protocols or clinical practice.

Novel techniques providing direct imaging of lipid distributions and dynamics in blood vessels and tissues could enable non-invasive tests that evaluate the risk for CVD and metabolic diseases, as well as the easy monitoring of nutritional and other metabolic conditions that are difficult to study. Here, we aimed to introduce a method that could go beyond the current state of the art in measuring postprandial lipid dynamics by satisfying three critical specifications. First, the method should be safe, non-invasive and portable, so that it can be seamlessly disseminated to studies of large populations. Second, it should be capable of recording lipid measurements in different tissue compartments, not only single-point bulk measurements, enabling differential studies of lipid circulation and uptake in tissues of interest. Third, it should be capable of frequent sampling to provide a detailed profile of highly resolved spatio-temporal lipid dynamics. Introducing such functionality into lipid research and medical care could significantly expand our knowledge of individual responses to nutritional challenges and offer new abilities for cardiovascular and metabolic risk assessment on a personalized basis.

**Figure 1:**
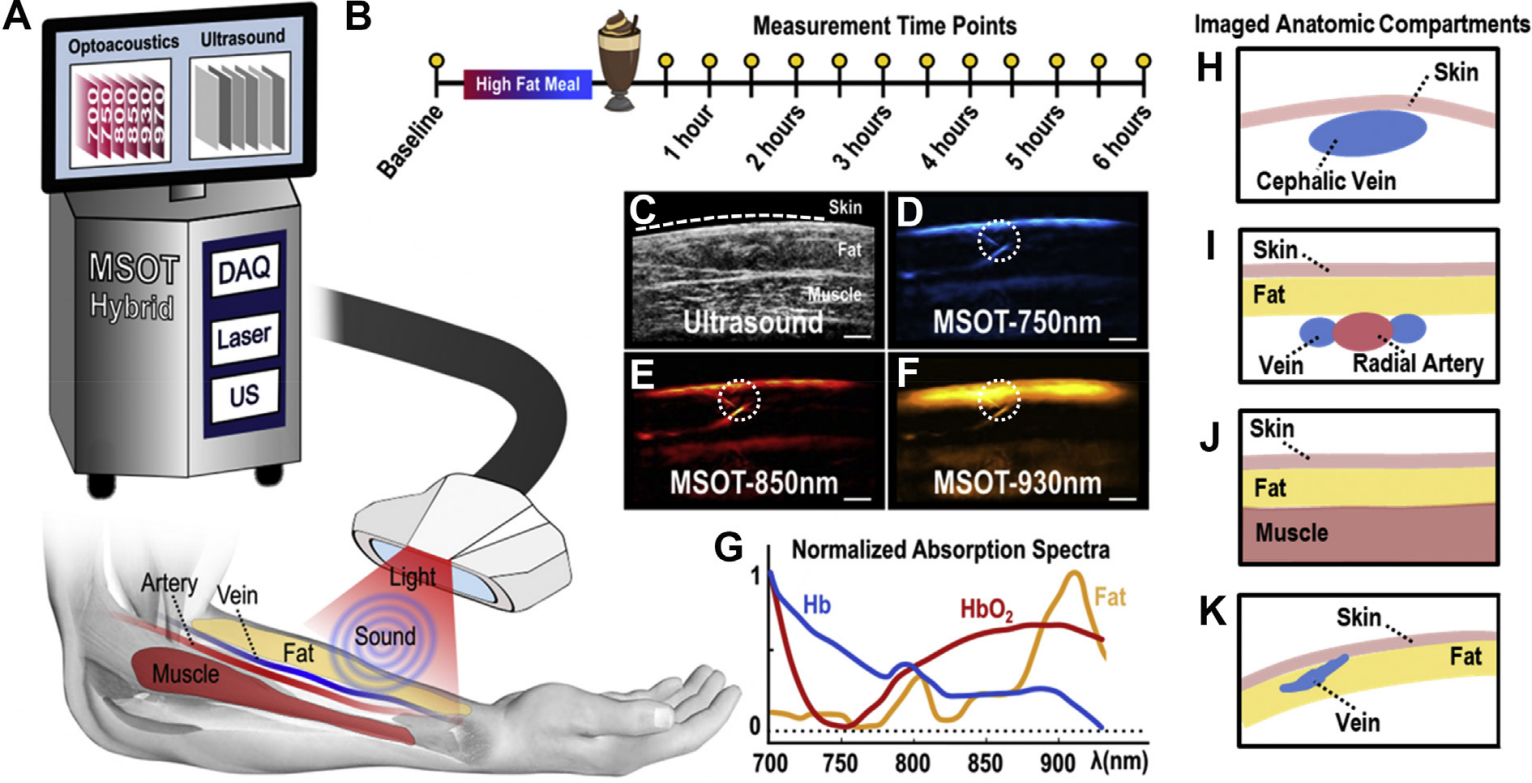
MSOT principle of operation and study design. (A) Configuration of the clinical hybrid MSOT/US system. (B) Postprandial lipemia measurement protocol. (C) Exemplary ultrasound image where the skin line (white dashed line), the subcutaneous fat and the skeletal muscle areas are shown. (D–F) MSOT images corresponding to the ultrasound image in (C). The dotted white circles show a small vessel detail within the subcutaneous fat region, demonstrating the excellent resolution performance of clinical MSOT technology. (D) MSOT image at 750 nm, representing mainly the distribution of deoxygenated Hb. (E) MSOT image at 850 nm, representing mainly the distribution of oxygenated Hb. (F) MSOT image at 930 nm, representing mainly the spatial distribution of lipids. Scale bars are 0.5 cm. (G) Absorption spectra in the near-infrared range (NIR) for Hb, HbO_2_ and fat/lipids. (H–K) Schematic diagrams of imaged anatomic compartments in the human forearm. (H) Cephalic vein. (I) Radial artery. (J) Skeletal muscle. (K) Subcutaneous fat.

To introduce this paradigm-shifting performance, we hypothesized that optoacoustic imaging, in particular multispectral optoacoustic tomography (MSOT), could offer a platform to obtain localized non-invasive sensing of lipid concentrations. MSOT acquires images of tissue molecules exhibiting optical absorption at multiple wavelengths in the near-infrared range (NIR) and resolves spectral information, revealing deoxygenated hemoglobin (Hb), oxygenated hemoglobin (HbO_2_), and lipids among others (Figure 1) [15]. Thus, MSOT should enable the monitoring of lipid distributions by means of wavelength selection within different tissue compartments. Preliminary evidence demonstrated that MSOT can resolve blood vessels, skeletal muscle, and adipose tissues [[16], [17], [18], [19], [20], [21], [22]]. Furthermore, it was recently shown that MSOT can visualize oxidative metabolism by monitoring the rate of conversion of oxygenated hemoglobin to deoxygenated hemoglobin, revealing oxygen utilization by tissue [16,23,24]. More specifically, observations in animals were corroborated with measurements of oxidative metabolism during brown fat activation in humans and mice [16,23,24]. However, it is currently unknown whether MSOT can resolve lipid dynamics in response to nutritional inputs. Therefore, we employed MSOT to visualize vasculature and other soft tissue compartments in the 700–970 nm spectral window. Then, using observations at 930 nm, a wavelength in which lipids exhibit an absorption peak in the near-infrared [25], we investigated whether we could resolve lipid-specific signals over time in different tissue compartments in response to oral fat intake. MSOT is a safe modality that uses light, does not require labels for imaging lipids and offers high resolution (<300 μm) and large fields of view (>4 cm) while reaching depths of 3–4 cm in living tissue. We demonstrate in this study that these capabilities combine to afford a novel tool for the non-invasive study of lipid dynamics.

## 2 Materials and methods

### 2.1 Study design and experimental protocol

Four (n = 4) subjects (3 females and 1 male, age: 28 ± 7 years) with body mass indices (BMI in kg/m^2^) of 28 (subject 1), 31 (subject 2), 23 (subject 3), and 21 (subject 4) were enrolled in the current pilot study. All volunteers were non-smokers and had no history of cardiovascular or metabolic disease. They were kindly requested to avoid consuming caffeine, food, and alcohol for at least 12 h before the planned measurements. Signed informed consent was obtained from all participants prior to study enrollment. The study was approved by the ethics committee of the medical faculty of the Technical University Munich (Protocol No 349/20 S).

Measurements were conducted in a normal examination room at a temperature of 23 °C. After a 12-h overnight fast, participants ingested an oral fat load within 5 min. The consistency of the fatty meal was 350 ml of pasteurized heavy cream, 15 ml of fat-free milk, 15 ml of chocolate syrup, and 1 tablespoon of granulated sugar. This fat load contained 117 g of fat (70 g saturated fat, 467 mg cholesterol), 41.5 g of carbohydrate, and 0.5 g of protein and provided 1,242 calories (86.4% from fat, 13.4% from carbohydrates, 0.2% from protein) [26]. The high-fat liquid meal was assigned to simulate the fat content of a typical high-fat meal.

A scanning probe was repeatedly placed in the same positions over the radial artery, the cephalic vein, and the brachioradialis muscle of the dominant forearm, guided by stable skin markers (Figure 1A). Recordings of these regions were taken post-fasting and then every 30 min and for 6 h after consumption of the fatty meal (13 measurements in total), which was determined to be a suitable timespan to assess postprandial responses (Figure 1B). The fourth participant felt some light gastric disturbances; hence, we decided to acquire less measurements and stop the experiment at 5 h after the oral loading (5 measurements in total). Each anatomic compartment was scanned for 10 s.

Apart from prior anatomical knowledge, the arteries were initially identified from their pulsation, the veins from their compressibility with the hand-held probe, and the subcutaneous fat and the skeletal muscles by their characteristic textures in traditional ultrasound (US) (Figure 1C). The identification of the different tissues and anatomical compartments was further facilitated by means of their MSOT appearance: the blood vessels and skeletal muscles were characterized by an increased absorption at the 750 nm and 850 nm, compared to the absorption at 930 nm, due to the strong presence of the Hb and HbO_2_ in these tissue compartments and the prominent absorption of both at these NIR-wavelengths [19]. Correspondingly, the subcutaneous fat tissue was characterized by an absorption peak at 930 nm where lipids absorb the most in the NIR (Figure 1D–G).

### 2.2 MSOT data acquisition

Measurements were conducted using a hybrid clinical MSOT/US (Acuity©, iThera Medical GmbH, Munich Germany). For ultrasound detection, the hand-held probe (Figure 1A) was equipped with 256 piezoelectric elements with a central frequency of 4 MHz arranged in an arc of 145°. Illumination was achieved through an optical fiber, mounted on the same hand-held probe. Light was emitted in the form of short pulses (10 ns in duration), at a rate of 25 Hz. For each pulse, almost 15 mJ of energy were delivered over a rectangle area of around 1 × 4 cm, ensuring compliance with the safety limits of laser use for medical applications [27]. For multispectral image acquisition, we employed 28 different light wavelengths (from 700 to 970 nm at steps of 10 nm). Thus, the recording of one ‘multispectral stack’, or a full set of 28 single-wavelength optoacoustic images, lasted 1 s. Co-registered US images were recorded in parallel to the MSOT images at frame rate of 8 Hz.

As previously shown, MSOT images acquired at 750 nm reveal primarily Hb contrast, whereas images at 850 nm reveal contrast primarily from HbO_2_ [19]. MSOT images at the 930 nm show mainly tissue lipid distribution [16,20,25]. Thus, observed differences in the informational content, or else the appearance of different tissues, in the abovementioned single-wavelength MSOT images (Figure 1D–F) are based on: i) the different features of the known absorption spectra of Hb, HbO_2_ and lipids in the NIR (Figure 1G) [25] and ii) the content of Hb, HbO_2_ and lipids in the different tissues of each anatomic compartment.

The appearance of different tissues in the MSOT images (Figure 1D–F) showed good spatial correspondence to the co-registered US images (Figure 1C), but with additional functional and molecular contrast. Thus, illumination at 930 nm (Figure 1F) highlights the subcutaneous fat region, which mainly contains lipids and is therefore characterized by much stronger light absorption or else optoacoustic image intensity, compared to adjacent blood vessels and muscles. The dotted white circles in Figure 1D–F mark a small blood vessel in the subcutaneous fat region, highlighting the details that can be recorded by means of clinical MSOT, which achieves a spatial resolution of less than 300 μm.

Figure 1H–K shows the anatomical compartments, which were selected for analysis in MSOT images recorded at 930 nm to sense postprandial lipid dynamics in a variety of tissues: i) venous and arterial blood, the gold standard tissues employed for quantifying lipid dynamics in clinical practice, ii) skeletal muscle, which promotes the easy intra-tissue distribution of lipids via its high vascularization and the high-contrast blood lipid imaging due to its low lipid content under normal conditions (best-case scenario) and iii) subcutaneous fat, which is poorly vascularized compared to muscle, and provides a low-contrast environment for postprandial lipemia imaging due to its high lipid content (worst-case scenario).

### 2.3 Data processing and analysis

Acquired MSOT data were reconstructed using a model-based reconstruction method [28]. The blood vessels (radial artery and cephalic vein) and the soft tissues of the forearm (subcutaneous fat and muscle) were first identified in consensus between two clinicians with experience in clinical MSOT and ultrasound imaging. The identification was based on anatomical knowledge, ultrasound guidance, and the characteristic MSOT appearance for each tissue. For each subject, a set of characteristic 930 nm-frames for all measurement time points was selected. Next, precise regions of interest (ROIs) within the arterial or venous lumen, the subcutaneous fat, and the muscle regions were manually segmented in consensus with two independent clinicians with experience in clinical MSOT and ultrasound imaging. The agreement between the two groups of ROIs was high, as indicated by the Cohen's Kappa value (mean 0.945, interquartile range 0.921–0.969), supporting the reliability of the segmentation process. Finally, the mean intensity values of the pixels belonging to the manually-segmented ROIs were used to plot the time course of the MSOT-extracted lipid signals within each compartment during the lipemia challenge (*see Results*). Lipid signal calculations took place on the recorded 930-nm images, without the application of any filtering or denoising. Calculated values were reported as means or means ± standard deviation.

## 3 Results

**Figure 2:**
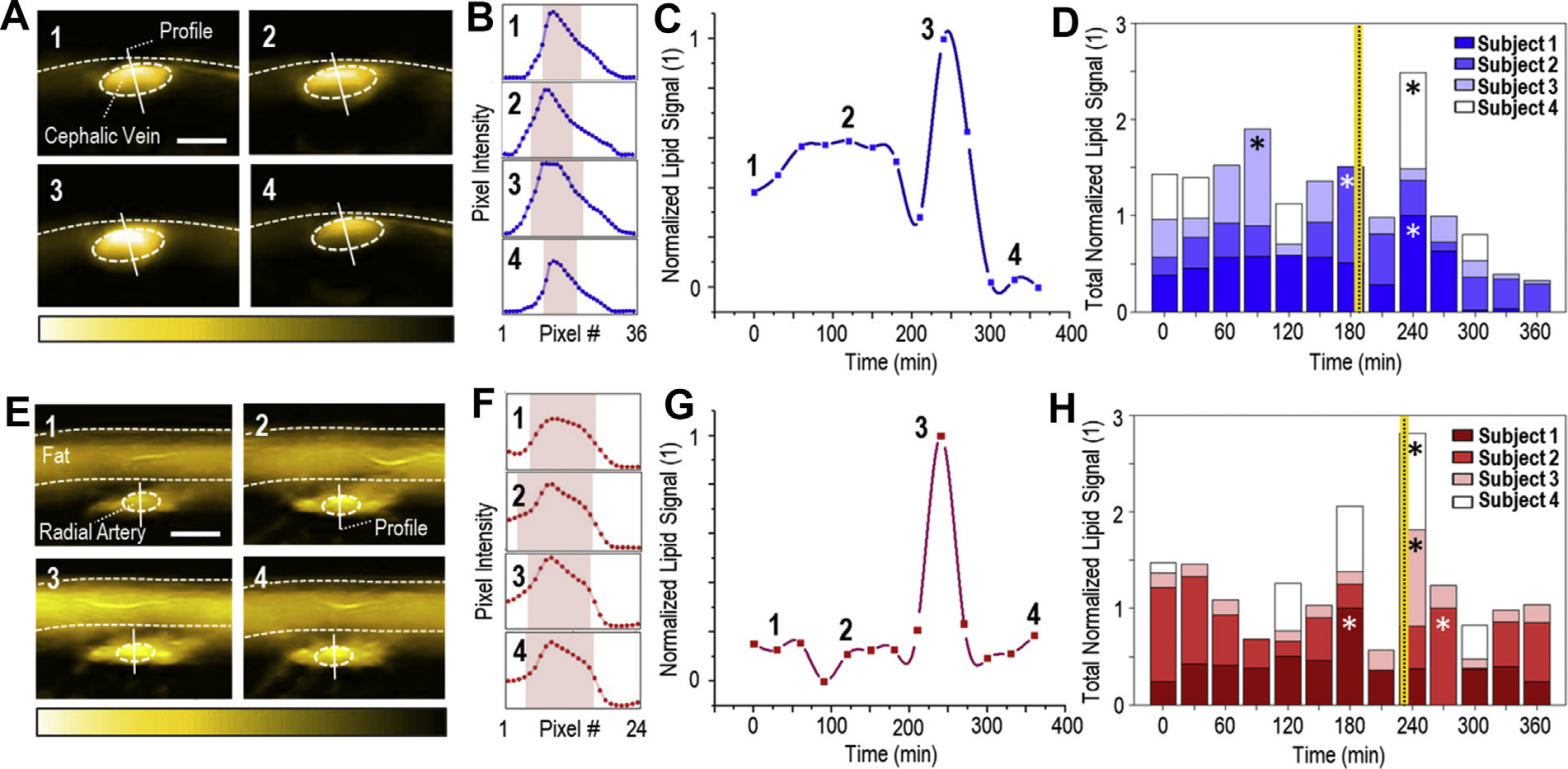
MSOT imaging of postprandial lipid dynamics in the blood of veins and arteries. (A) A series of cross-sectional MSOT images of the cephalic vein recorded at 930 nm, which correspond to the four time points indicated in (C) (subject 1). White dashed line: skin surface. White dashed ellipse: cephalic vein. Scale bars: 0.4 cm. (B) Pixel-intensity cross-sections along the corresponding profile lines in the image series of Figure 2A. The red bands show the pixel range at 50% of the maximum pixel-intensity value along the profile line. (C) Normalized mean lipid signal within the cephalic vein for subject 1 during the whole postprandial lipemia test. The first time point corresponds to the fasting state. (D) MSOT-extracted lipid dynamics for the cephalic veins of all four subjects. The asterisks indicate the time points of the maximum-recorded value for each subject. The vertical yellow-black line indicates the average time point (among all subjects) after oral loading for the maximum-recorded lipid signal within the vein. (E) Cross-sectional MSOT images (at 930 nm) of the radial artery for the four time points of (G) (subject 3). Upper white dashed line: skin surface. Lower white dashed line: lower limit of the subcutaneous fat region. White dashed ellipse: radial artery. Scale bars: 0.3 cm. (F) Pixel-intensity cross-sections along the profile lines of Figure 2E-image series. Red bands: pixel range at 50% of the maximum pixel-value along the profile line. (G) Postprandial lipid dynamics (normalized) within the radial artery for subject 3. (H) MSOT-extracted lipid dynamics for the radial arteries of all subjects. Asterisks: time point of the maximum-recorded value for each subject. The vertical black-yellow line indicates the average recorded time point for the maximum lipid signal within the artery to be recorded.

**Figure 3:**
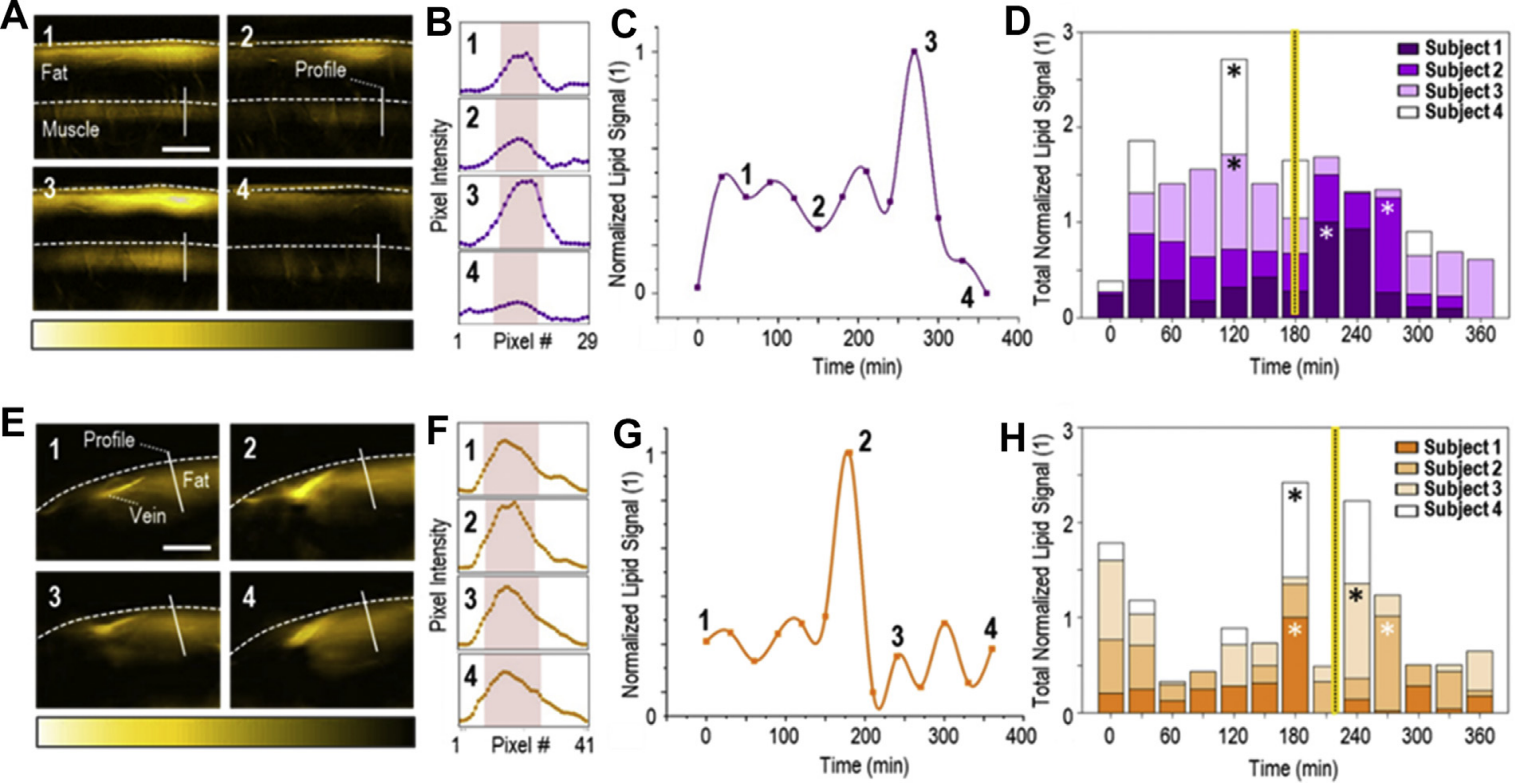
MSOT imaging of lipid dynamics in skeletal muscle and subcutaneous fat. (A) A series of cross-sectional MSOT images of the brachioradialis muscle recorded at 930 nm, which correspond to the four time points indicated in (C) (subject 2). Upper white dashed line: skin surface. Lower white dashed line: upper limit of the muscle region. Scale bars: 1 cm. (B) Pixel-intensity cross-sections along the corresponding profile lines in the image series of Figure 3A. The red bands show the pixel range at 50% of the maximum pixel-intensity value along the profile line. (C) Normalized mean lipid signal within the muscle for subject 2 during the whole postprandial lipemia test. The first time point corresponds to the fasting state. (D) MSOT-extracted lipid dynamics for the brachioradialis muscles of all four subjects. The asterisks indicate the time points of the maximum-recorded value for each subject. The vertical yellow-black line indicates the average time point (among all subjects) after oral loading for the maximum lipid signal within the muscle to be recorded. (E) Cross-sectional MSOT images (at 930 nm) of the forearm subcutaneous fat for the four time points of (G) (subject 1). Upper white dashed line: skin surface. Scale bars: 0.5 cm. (F) Pixel-intensity cross-sections along the profile lines of Figure 3E-image series. Red bands: pixel range at 50% of the maximum pixel-value along the profile line. (G) Postprandial lipid dynamics (normalized) within the subcutaneous fat region for subject 1. (H) MSOT-extracted lipid dynamics for the subcutaneous fat of all subjects. Asterisks: time point of the maximum-recorded value for each subject. The vertical black-yellow line indicates the average recorded time point for the maximum lipid signal within the subcutaneous fat to be recorded.

We applied MSOT to study postprandial lipid dynamics in the blood of the cephalic vein (Figure 2A–D) and the radial artery (Figure 2E–H), as well as, in soft tissues (skeletal muscle, Figure 3A–D and subcutaneous fat, Figure 3E–H). Figure 2A illustrates a series of characteristic MSOT images of a cross-section of the cephalic vein (subject #1) acquired at 930 nm that correspond to four time points: before oral loading (1), and 120 min (2), 240 min (3), and 330 min (4) after oral loading. The 3rd image (240 min postprandially) corresponds to the time point of the maximum intravenous lipid content. We also analyzed the pixel intensities along the profile lines of the previous image series to gain insights into the contrast between the lipid signal detected inside the current ROI and that of adjacent structures (Figure 2B), that may be also affected during postprandial lipemia. Our results show that both the spatial and temporal fluctuations in lipid signals inside the structures of interest are clearly higher compared to adjacent structures, proving that the observed phenomena are not caused by random fluctuations of the 930-nm signal over the whole image, but by a postprandial increase in the venous blood.

Figure 2C illustrates the changes in the mean lipid signals within the cephalic vein over the whole duration of the experiment for subject #1. Plotted mean values were normalized against the maximum mean value (at 240 min), with the numbered data points corresponding to the MSOT images in Figure 2A. The first time point of Figure 2C represents the baseline state. The lipid signal in the cephalic vein of subject #1 reached its highest value (+32.1% compared to subject's baseline) 240 min after the consumption of the meal (Figure 2C, point 3). Figure 2D shows the normalized lipid signals recorded within the cephalic veins of all subjects for each time point. For Subject #2, the highest value was recorded at 180 min postprandially (+170% compared to the subject's baseline). Subject #3 shows a maximum lipid value of +95.1% compared to baseline at 90 min after meal consumption. The highest MSOT-measured lipid signal in the cephalic vein of Subject #4 is observed at 240 min postprandially (+38.5% compared to subject's baseline). In summary, a mean maximum increase of +83.9% is reported among the four subjects. The intra-venous lipid signal reaches its maximum value on average 187.5 min (3 h) after the oral loading. All percentages were extracted from the measured, and not the normalized, optoacoustic signal values. A detailed description of the maximum optoacoustically-extracted lipid values for all subjects and measured anatomic compartments is provided in Table 1.

**Table 1:** Postprandial time points of the maximum-recorded lipid signal within the segmented anatomic compartments (cephalic vein, radial artery, skeletal muscle, and subcutaneous fat) for all subjects, as compared to the baseline.

Anatomic Compartment	Subject #	Time of Peak (min)	Relative Increase (%)
Cephalic Vein	1	240	32.1
	2	180	170
	3	90	95.1
	4	240	38.5
	**Means**	**187.5**	**83.9**
Radial Artery	1	180	46.4
	2	270	0.9
	3	240	69.3
	4	240	136.9
	**Means**	**232.5**	**63.4**
Skeletal Muscle	1	210	49
	2	270	170.8
	3	120	145.5
	4	120	117.7
	**Means**	**180**	**120.8**
Subcutaneous Fat	1	180	39.7
	2	270	25
	3	240	11.3
	4	180	55.2
	**Means**	**217.5**	**32.8**

In brief, the outcomes of the radial artery are similar (Figure 2E–H), with the highest intra-arterial lipid value being recorded at 232.5 min (4 h) after the oral loading, on average. The mean maximum increase observed is 63.4%, relative to the baseline. Furthermore, soft tissues also exhibited a prominent peak in postprandial lipids (Figure 3). An average time span of 180 min is needed for the lipid signal to reach its maximum within the skeletal muscle, which corresponds to an increase of 120.8% relative to baseline (Figure 3A–D). Finally, for the subcutaneous fat, the mean maximum increase is 32.8%, detected on average 217.5 min (4 h) after the oral loading (Figure 3E–H).

To further investigate the observed variability of the postprandial time points of the maximum-recorded lipid signal, we performed a more elaborated statistical analysis with reference to the: i) different anatomic compartments examined and ii) different BMIs of the subjects. Even considering the small sample size, we observed that the anatomic compartment with the lowest standard deviation of the recorded maximum-value time points was the radial artery (232.5 ± 37.7 min). Furthermore, we observed that the two subjects (subjects 1 and 2) characterized as overweight (BMI > 25 kg/m^2^) showed lower standard deviations (subject 1: 28.7 min, subject 2: 45 min, subject 3: 78.9 min, subject 4: 57.4 min) of the maximum-value time points throughout the examined anatomic compartments.

## 4 Discussion

Postprandial lipemia is a dynamic condition characterized by an increase in blood lipid levels after the consumption of a meal compared with relatively stable fasting conditions. Pathological postprandial lipid profiles, in particular prolonged high lipid levels in blood, have been associated with serious diseases, such as diabetes, obesity, and CVD [29]. Thus far, fluctuations of lipid levels in the blood stream have been monitored either by traditional blood sampling, or with non-invasive, but indirect, methods. We have demonstrated herein that a hand-held MSOT can non-invasively visualize and quantify lipid fluctuations in human blood vessels and soft tissues for several hours after the ingestion of a high-fat meal. This technique offers two key advantages over other non-invasive methods for *in vivo* lipid measurements: i) it provides direct lipid-specific molecular information in blood vessels and soft tissues without the need for injected contrast agents; and ii) it has excellent spatial resolution of less than 300 μm for detailed tomographic imaging of *in vivo* lipid distributions over time.

The results of our pilot study demonstrate that MSOT can provide time-resolved data on lipid levels during the postprandial period. Light absorption by lipids reaches its peak in the NIR at 930 nm, and signals recorded by MSOT in tissue upon illumination at this wavelength have been strongly associated with lipid content [25]. Our recordings showed a clear increase in signal intensities within the segmented anatomical compartments of interest (cephalic vein, radial artery, brachioradialis muscle, and subcutaneous fat) approximately 3–4 h on average after the subjects consumed a fatty meal.

Furthermore, MSOT provides direct molecular imaging of lipid dynamics within different anatomic compartments, most importantly within blood, the gold standard for biochemical lipid analysis in clinical settings. Other image-based methods, such as eye image analysis [14], provide an indirect estimation of the circulating lipids based on the lipid deposits on the cornea. The presented analysis of the recorded color images is based on the mean grayscale intensity within the segmented corneal ROI: a feature that may be affected by the ambient light and hinder the image-based differential diagnosis of corneal lipid depositions from other conditions, such as the limbus sign, which indicates calcium and not lipid deposits in the cornea [30]. Nevertheless, the segmentation approach that has been used for the iris outer boundary by means of circle detection [14] could indeed be used for the automatic segmentation of blood vessels in MSOT images [31], a development which is expected to significantly facilitate the analysis process, especially for large datasets. Such an implementation could take place via a more generalized approach by searching for elliptical objects, which largely resemble the shape of vascular cross-sections in tomographic images. Moreover, an ellipse fitting approach for tracking the wall dynamics in the radial artery has already been applied on MSOT data, with good results [18]. Finally, more elaborate statistical and multidimensional image analysis approaches could be applied to both techniques to reveal possibly correlations between the features extracted in MSOT images and the ones extracted in eye images with regard to postprandial lipemia or relevant conditions, such as the hypercholesterolemia.

The results agree with known postprandial lipid patterns recorded by blood collection [29] and demonstrate the unique capability of MSOT to provide direct and non-invasive monitoring of in *vivo* lipid level fluctuations in the postprandial state without the need for contrast agents. Moreover, although we only acquired data every 30 min for the current study, the high temporal resolution of MSOT (25 Hz) would enable the non-invasive investigation of faster phenomena of lipid kinetics to be explored in future studies.

MSOT also provided high-resolution visualizations of lipids in blood vessels and soft tissues simultaneously, which is enabled by its 2- to 4-cm depth-penetration and approximately 4-cm horizontal field of view. Thus, using MSOT, we were able to detect tissue variations in the occurrence of the highest average postprandial signals. The most intense signals in the cephalic vein and the skeletal muscle were recorded at ~3 h, while the corresponding signals in the radial artery and the subcutaneous fat were recorded at ~ 4 h. To our knowledge, such rich and direct information on lipid dynamics has not been provided before in the literature. The ability to image both the blood circulation (arteries and veins) and soft tissues (adipose tissue and muscle) during dynamic phenomena, such as the postprandial lipemia, may reveal metabolic interactions among the involved compartments and tissue components. Thus, MSOT could ideally help recognize paths that regulate the crosstalk between cardiovascular and metabolic components of human physiology and pathophysiology of diseases, such as obesity, hypertension, and diabetes [32].

The current study introduces MSOT as a powerful tool for the non-invasive monitoring of *in vivo* blood lipid dynamics during the postprandial state. Our results open up new possibilities in the diagnostics and risk assessment of CVD and metabolic disease, especially when the high portability and low complexity of hand-held MSOT is expected to further facilitate its future disseminated use. Furthermore, the unique capability of MSOT technology to provide real-time and label-free visualizations of intra-vascular, intra-muscular, and intra-subcutaneous fat lipid maps renders it an ideal tool for basic and clinical research in the cardio-metabolic field.

The imaging depth of MSOT (2–4 cm) is excellent compared to other optical techniques, but is limited compared to traditional clinical modalities, such as ultrasonography. Nevertheless, in our study, we were able to access key blood vessels and soft tissues within these depth constraints and provide rich information on lipid dynamics in agreement with literature. The consideration of novel light fluence correction models that compensate for intensity attenuation due to scattering and absorption is expected to further improve the precision of MSOT imaging deeper in tissue and widen the range of clinical applications. Furthermore, the development of phantoms and advanced spectral unmixing algorithms [33] will facilitate the direct quantification of lipid concentrations deep within muscle or other soft tissues, rather than only their relative fluctuations. Our aim here was to demonstrate a proof-of-concept via a human pilot study with a small number of healthy participants. More extended studies including larger cohorts of healthy volunteers and patients, and simultaneous blood analyses are needed to further refine the application MSOT to the non-invasive monitoring of lipids.

Most individuals consume at least three meals per day, and each meal is usually consumed before the postprandially high blood lipid levels return to baseline. Consequently, individuals are in a postprandial state for approximately 18 h per day. Thus, the thorough investigation of *in vivo* lipid dynamics with novel methods may give new insights in several fields of basic and clinical cardio-metabolic research. It has been already shown that MSOT can provide precise anatomic, functional and molecular imaging of the vasculature and other soft tissues, such as adipose tissue and skeletal muscles [15,16,18,19]. This unique set of capabilities may facilitate the exploration of hidden mechanisms of cardio-metabolic crosstalk by enabling multifaceted investigations of common cardiovascular and metabolic diseases, such as atherosclerosis, diabetes, and lipid disorders. Further studies will advance hand-held MSOT toward its clinical translation with implications for objective diagnostics and therapy evaluation under patient- and operator-friendly conditions.

## 5 Conclusion

Clinical hand-held MSOT provides great potential to study lipid metabolism in the postprandial state. This unique feature could lead to novel diagnostics and prevention strategies by the label-free and non-invasive detection of lipid-related tissue biomarkers implicated in several cardiometabolic diseases.

## Author contributions

Study design: A.K., N.A.F. and V.N.; Measurements: A.K., N.A.F., A.B.M.; Manuscript preparation: all authors; Supervision: J.P., H.H.E., Mi. K., Ma.K., and V.N.

## Conflict of interest

V. Ntziachristos has stock/stock options in iThera Medical GmbH. All other authors have no conflicts of interest to declare.
